# Prostate-specific membrane antigen positron emission tomography (PSMA-PET) for local staging of prostate cancer: a systematic review and meta-analysis

**DOI:** 10.1186/s41824-020-00085-9

**Published:** 2020-09-09

**Authors:** Sungmin Woo, Soleen Ghafoor, Anton S. Becker, Sangwon Han, Andreas G. Wibmer, Hedvig Hricak, Irene A. Burger, Heiko Schöder, Hebert Alberto Vargas

**Affiliations:** 1 Department of Radiology, Memorial Sloan Kettering Cancer Center, 1275 York Avenue, New York, NY 10065 USA; 2 Department of Nuclear Medicine, Asan Medical Center, University of Ulsan College of Medicine, 88 Olympic-ro 43-gil, Songpa-gu, Seoul, 05505 Korea; 3 Department of Nuclear Medicine, University Hospital Zürich, University of Zürich, Zürich, Switzerland; 4 Department of Nuclear Medicine, Kantonsspital Baden, Baden, Switzerland

**Keywords:** Prostate-specific membrane antigen, Positron emission tomography, Prostate cancer, Computed tomography, Magnetic resonance imaging, Meta-analysis

## Abstract

**Purpose:**

Prostate-specific membrane antigen positron emission tomography (PSMA-PET) has shown promise for detecting nodal and distant prostate cancer (PCa) metastases. However, its performance for local tumor staging is not as well established. The purpose of this study was to review the diagnostic performance of PSMA-PET for determining seminal vesical invasion (SVI) and extraprostatic extension (EPE).

**Methods:**

Pubmed and Embase databases were searched until January 12, 2020. Studies assessing accuracy of PSMA-PET in determining SVI and EPE were included. Study quality was evaluated with the revised Quality Assessment of Diagnostic Accuracy Studies-2 tool. Pooled sensitivity and specificity were calculated using hierarchical summary receiver operating characteristics modeling. Heterogeneity was explored using meta-regression analyses for anatomical imaging component (MRI vs CT) and by testing for a threshold effect.

**Results:**

Twelve studies (615 patients) were included. Pooled sensitivity and specificity were 0.68 (95% CI 0.53-0.81) and 0.94 (95% CI 0.90-0.96) for SVI and 0.72 (95% CI 0.56-0.84) and 0.87 (95% CI 0.72-0.94) for EPE. Meta-regression analyses showed that for SVI, PET/MRI demonstrated greater sensitivity than PET/CT (0.87 [95% CI 0.75-0.98] vs 0.60 [95% CI 0.47-0.74]; *p* = 0.02 for joint model) while specificity was comparable (0.91 [95% CI 0.84-0.97] vs. 0.96 [95% CI 0.93-0.99]) but not for EPE (*p* = 0.08). A threshold effect was present for studies assessing EPE (correlation coefficient = 0.563 [95% CI, −0.234-0.908] between sensitivity and false-positive rate).

**Conclusion:**

PSMA-PET has moderate sensitivity and excellent specificity for assessing local tumor extent in patients with PCa. PET/MRI showed potential for greater sensitivity than PET/CT in assessing SVI.

## Introduction

Prostate cancer is the second most common cancer and the 5th leading cause of cancer-related deaths worldwide (Bray et al. 2018). Local staging and identification of nodal and distant metastases are important in determining the most appropriate management strategy. In surgical candidates planning to undergo radical prostatectomy, interrogating for the presence of seminal vesical invasion (SVI) and extraprostatic extension (EPE) is key, as they are associated with adverse oncological outcomes such as biochemical recurrence, metastasis, and worse survival (Mikel Hubanks et al. 2014). In addition, patients without EPE can undergo nerve-sparing surgery with the aim of reducing postoperative functional morbidity including urinary incontinence and erectile dysfunction (Mottet et al. 2017).

Nomograms combining clinicopathological information including prostate-specific antigen (PSA) levels, clinical stage based on digital rectal examination, and biopsy-related information (Gleason score, number, and percentage of positive cores) are often used to predict the extent of prostate cancer (Ohori et al. 2004). However, there is an increasing number of studies showing that incorporating preoperative magnetic resonance imaging (MRI) results provide incremental value in predicting SVI and EPE (Ohori et al. 2004; Nyarangi-Dix et al. 2018; Mehralivand et al. 2019; Park et al. 2020). Nevertheless, these results are still imperfect with area under the curves (AUC) ranging from 0.74-0.87 (Jansen et al. 2019; Wang et al. 2007; Weaver et al. 2018). A meta-analysis including over 9700 patients confirmed that the sensitivity of MRI for SVI and EPE is limited and heterogeneous among different studies with 57% (confidence interval (CI) 0.49-0.64) and 58% (CI 0.47-0.68), respectively. Therefore, there is an unmet clinical need to improve preoperative risk assessment in patients with prostate cancer.

Prostate-specific membrane antigen (PSMA) positron emission tomography (PET) is a relatively novel imaging technique, which targets PSMA, a transmembrane protein expressed on prostate cells with levels of expression increasing with greater degree of dysplasia (Bostwick et al. 1998; Hofman et al. 2018). Over the past few years, evidence has accumulated regarding the utility of PSMA-PET, especially those using ^68^Gallium (^68^Ga)-based radioligands. It is now recognized that this novel imaging modality is excellent in determining sites of disease in the biochemically recurrent post-treatment setting, identifying lymph node and bone metastases, and even in detecting the dominant lesion for primary staging, with these translating to actual impact in the management of patients (Fendler et al. 2019; Eiber et al. 2016; Perera et al. 2020; Zhou et al. 2019; Corfield et al. 2018; Han et al. 2018). However, the diagnostic performance of PSMA-PET in determining local disease extent is not well established as there are only scattered small-scaled reports in the literature. Therefore, the purpose of this study was to systematically review the literature and meta-analyze the diagnostic performance of PSMA PET for determining SVI and EPE based on radical prostatectomy as the reference standard.

## Materials and methods

### Search strategy and study selection

This study was performed according to the Preferred Reporting Items for Systematic Reviews and Meta-Analyses (PRISMA) guidelines (Liberati et al. 2009). Pubmed and Embase databases were systematically searched from inception until January 12, 2020, using keywords and related terms of “prostate”, “PSMA-PET”, “SVI”, and “EPE” based on the search query as the following: (prostate OR prostatic) AND (“prostate-specific membrane antigen” OR PSMA) AND (“positron emission” OR PET) AND (“extracapsular extension” OR ECE OR “extraprostatic extension” OR EPE OR “seminal vesical invasion” OR SVI OR T3 OR T3a OR T3b OR ((local OR localized OR regional OR locoregional) AND (stage OR staging OR extent* OR invasion))). The reference lists of eligible articles were also scrutinized to further identify relevant articles. No language limitations were applied.

Studies were included based on “Patient, Index test, Comparator, Outcome, and Study design” (PICOS) criteria: (1) “patients” with prostate cancer presenting for primary staging; (2) PSMA-PET as “index test;” (3) radical prostatectomy as the “comparator” or reference standard; (4) SVI or EPE as the “outcome;” and (5) “study design” of clinical trials, prospective or retrospective cohort studies either published as original articles or conference abstracts. Of note, we planned to only meta-analyze studies assessing ^68^Ga-based radioligands as they are widely used and investigated in the literature.

Studies were excluded if they (1) included a small number of patients (< 10), (2) were of other publication types (e.g., review articles, letters, or editorials); (3) focused on other topics; (4) did not provide sufficient data to calculate 2 × 2 contingency tables with regard to sensitivity and specificity; or (5) had overlap in the study population. When overlap was present, we used the study with more comprehensive information required for meta-analysis.

The study selection process was performed by two independent reviewers (S.W. and S.G.) and discussion with a third reviewer (H.A.V.) was performed when there was disagreement.

### Data extraction and quality assessment

Relevant study-, clinicopathological-, and PET-related information were extracted and collated in Excel 2016 as follows: (1) study: first author, publication year, institution, period of enrollment, country of origin, study design (prospective vs. retrospective), and endpoint (SVI, EPE, or both); (2) clinicopathological: number of patients, age, serum PSA level, Gleason score, risk classification (Mottet et al. 2020), (3) PET: vendor, type of scanner, ligands, anatomical imaging component (MRI vs. CT), and whether PET was assessed blinded to clinicopathological information or not.

The quality of the studies was assessed using the revised Quality Assessment of Diagnostic Accuracy Studies-2 (QUADAS-2) tool (Whiting et al. 2011). Data extraction and quality assessment were performed by the same three reviewers above in the same manner.

### Data synthesis and analysis

The primary outcome of our study was to assess the diagnostic performance of PSMA-PET for determining SVI and EPE in terms of sensitivity and specificity. The secondary outcome was to evaluate whether there are differences in the performance between PET/MRI and PET/CT.

True positive, false negative, false positive, and true negative values were tabulated using sensitivity and specificity or the corresponding raw data provided from each of the included studies. If multiple diagnostic test accuracy results by multiple readers were given within a study, the average value across all readers was used. Sensitivity and specificity were meta-analytically pooled using hierarchical logistic regression modeling and corresponding hierarchical summary ROC (HSROC) curves were generated with their 95% confidence and prediction regions (Suh and Park 2016; Lee et al. 2015). Publication bias was evaluated by subjective assessment of the Deeks’ funnel plot and based on the *p* value of Deeks’ asymmetry test (Deeks et al. 2005).

Heterogeneity was assessed with several methods. First, heterogeneity was evaluated using the Cochran’s *Q* test. Second, Higgins *I*^2^ test was used to determine the degree of heterogeneity as follows: inconsistency index (*I*^2^) = 0–40%, unimportant; 30–60%, moderate; 50–90%, substantial; and 75–100%, considerable (Higgins and Green 2011). Third, we tested for the presence of a threshold effect, which means a positive correlation between the sensitivity and false-positive rate. Finally, meta-regression analysis was performed using anatomical imaging component of the PET (MRI vs. CT) as a covariate to ascertain if there were differences in the diagnostic performance between studies using PET/MRI and PET/CT.

The “metandi” and “midas” modules in Stata 10.0 (StataCorp LP, College Station, TX, USA) and “mada” package in the *R* software version 3.6.1 (R Foundation for Statistical Computing, Vienna, Austria) were used for statistical analyses. A two-tailed *P* < 0.05 was considered statistically significant with the exception of Deeks’ asymmetry test, where < 0.1 indicated statistical significance.

## Results

### Literature search

**Fig. 1:**
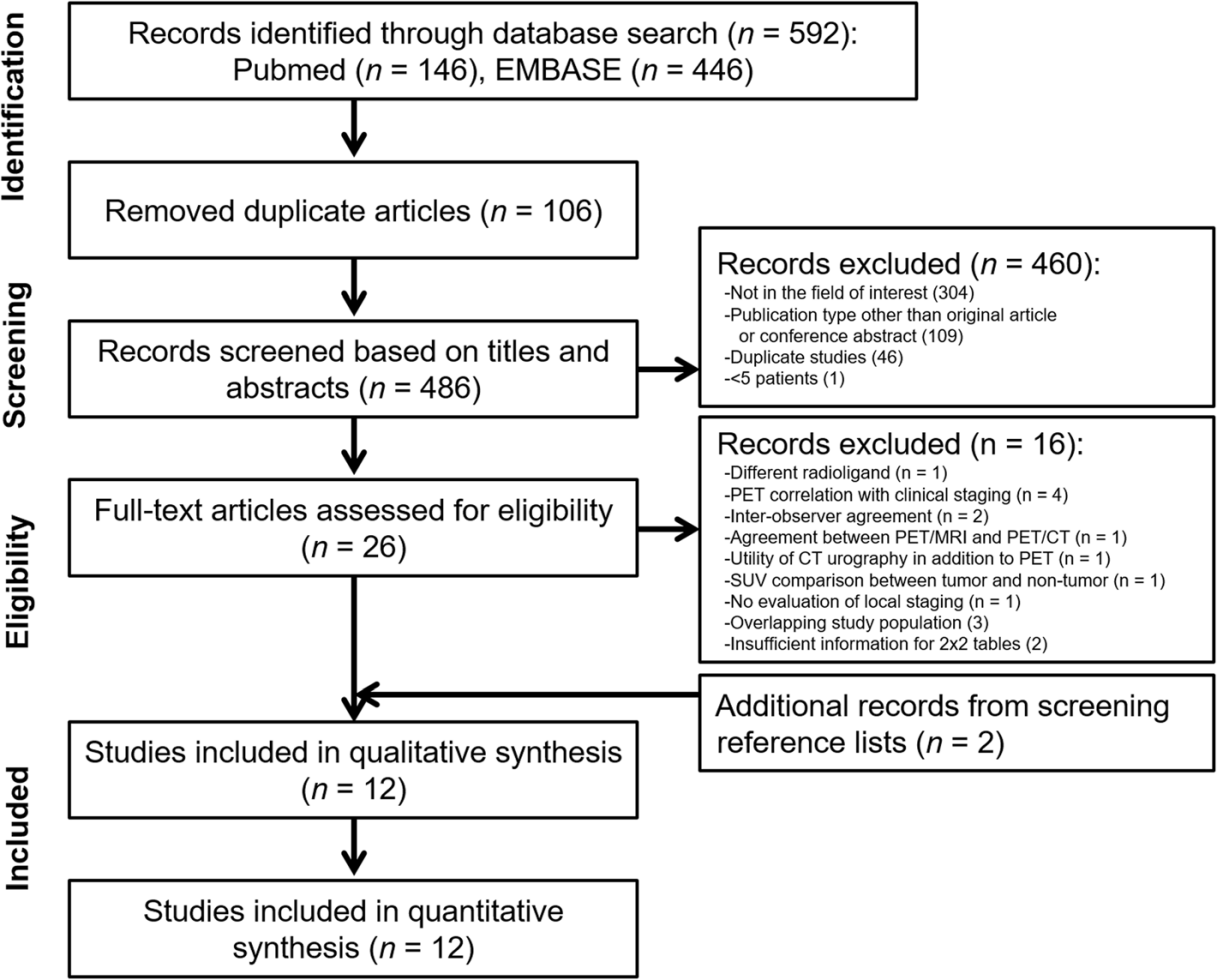
Flow diagram for study selection process

Initially, 592 articles were identified from the systematic search. After removal of 106 duplicates, 460 articles were further excluded by screening the titles and abstracts. Full-text reviews were done on the remaining 26 articles, among which 16 studies were excluded owing to the following reasons: non-^68^Ga-based radioligands (^18^F–PSMA-1007) was used (*n* = 1), PSMA PET was correlated with clinical staging (*n* = 4), inter-observer agreement study (*n* = 2), an agreement between PET/MRI and PET/CT (*n* = 1), assessment of utility of CT urography together with PET (*n* = 1), comparison of standardized uptake value between tumor and nontumor (*n* = 1), no evaluation of local staging (*n* = 1), insufficient data for reconstructing 2 × 2 tables (*n* = 2), and overlap in patient population (*n* = 3). Two additional articles were found upon additional screening of the reference lists. Finally, 12 studies including a total of 615 patients were included (Agrawal et al. 2017; Berger et al. 2018; Dekalo et al. 2019; Fendler et al. 2016; Gao et al. 2019; Grubmuller et al. 2018; Gupta et al. 2018; Muehlematter et al. 2019; Thalgott et al. 2018; van Leeuwen et al. 2019; von Klot et al. 2017; Yilmaz et al. 2019). All 12 assessed SVI while 8 of them evaluated EPE. The study selection process is shown in Fig. 1.

### Characteristics of included studies

The characteristics of the included studies are summarized in Tables 1 and 2. In brief, all studies were retrospective single-center studies except for one prospective single center (Grubmuller et al. 2018) and one retrospective dual center study (van Leeuwen et al. 2019). The number of patients ranged from 21 to 140 with median ages ranging from 63 to 70 years. Median PSA levels were 7.6-58.7 ng/mL and the median Gleason scores were 7-9. Two studies only included patients with high risk, 7 with intermediate to high risk, and 2 with low to high risk (which were predominantly constituted with intermediate to high-risk patients). Eleven studies used ^68^Ga-PSMA-11 and one used ^68^Ga-PSMA-I&T. Anatomical imaging was based on MRI in 3 studies and CT in 9.

**Table 1:** Study and Clinicopathological characteristics of 13 included studies

	Study characteristics						Clinicopathological characteristics				
	Origin			Design		Endpoint	No. of patients	Age (year)	PSA (ng/mL)	Gleason score	Risk group
Author, publication year	Institution	Country	Enrollment period	Prospective	Multicenter						
Agrawal A, 2017	TATA Memorial Hospital	India	NR	No	No	SVI	35	NR (47-73)	NR (NR)	NR (NR)	NR
Berger I, 2018	Nepean Hospital	Australia	Feb. 2015-Jan. 2017	No	No	SVI	50	64.9^a^ (± 5.6)	10.6^a^ (± 8.1)	7 [4 + 3] (6-9)	^b^Low, intermediate, high
Dekalo S, 2019	Tel-Aviv Sourasky Medical Center	Israel	NR	No	No	SVI	59	65.35* (± 6.99)	12.97^a^ (11.88)	7 [4 + 3] (7-9)	Intermediate, high
Fendler WP, 2016	Ludwig-Maximilians-University of Munich	Germany	Jan. 2014-April 2015	No	No	SVI, EPE	21	70.1^a^ (± 5.9)	58.7^a^ (± 85.2)	7 [4 + 3] (6-9)	High
Gao J, 2019	Drum Tower Hospital	China	Nov. 2017-Dec. 2018	No	No	SVI, EPE	49	69 (55-82)	15.94 (4.04-72.05)	7 [4 + 3] (7-8)	Intermediate, high
Grubmuller B, 2018	Medical University of Vienna	Austria	April 2014-July 2017	Yes	No	SVI, EPE	80	64 (59-71)	7.63 (5.5-13.4)	7 (6- > 8)	Intermediate, high
Gupta M, 2018	Rajiv Gandhi Cancer Institute and Research Centre	India	July 2014-March 2017	No	No	SVI, EPE	23	66 (38-88)	NR (≤ 10- > 20)	NR (6-10)	Intermediate, high
Muehlematter UJ, 2019	University Hospital Zurich	Switzerland	April 2016-July 2018	No	No	SVI, EPE	40	63^a^ (± 6)	8.12 (IQR 7.56)	8 (7-9)	Intermediate, high
Thalgot M, 2018	Klinikum Rechts der Isar	Germany	Dec. 2012-Nov. 2015	No	No	SVI, EPE	73	68 (63-73)	14.0 (6-35)	8 (6-10)	High
van Leeuwen PJ, 2019	St Vincent’s Hospital, Netherlands Cancer Institute	Australia, Netherlands	Feb. 2015-Oct. 2017	No	Yes	SVI	140	NR	9.4 (NR)	8 (7-9)	Intermediate, high
von Klot CAJ, 2017	Hannover Medical School	Germany	NR	No	No	SVI, EPE	21	68 (56-77)	11.9^a^ (NR)	7 [3 + 4] (6-10)	Intermediate, high
Yilmaz B, 2019	Istanbul Research and Training Hospital	Turkey	May 2016-April 2018	No	No	SVI, EPE	24	62.8^a^ (± 6.4)	12.0^a^ (± 7.4)	7 [4 + 3] (6-9)	^b^Low, intermediate, high

*CT* Computed tomography, *EPE* Extraprostatic extension, *MRI* Magnetic resonance imaging, *NR* Not reported, *PET* Positron emission tomography, *PSA* Prostate-specific antigen, *PSMA* Prostate-specific membrane antigen, *SVI* Seminal vesical invasion

^a^Data presented in mean ± standard deviation; others are presented in median and ranges

^b^Predominantly intermediate to high-risk patients as there were only 1 (at most) and 3 patients within the low-risk category in the studies by Berger et al. (2018) and Yilmaz et al. (2019), respectively

**Table 2:** PSMA-PET characteristics of 13 included studies

Author, publication year	Ligand	Scanner vendor/model	Mean dose (MBq)	Uptake time (min)	Acquisition time (min/bed)	Furosemide	Criteria for SVI or EPE	Blinded reading	Anatomical imaging	MRI details	Contrast-enhanced CT
Agrawal A, 2017	^68^Ga-PSMA-11	NR/NR	NR	NR	NR	NR	None	NR	CT		NR
Berger I, 2018	^68^Ga-PSMA-11	Philips/Gemini TF 64	NR	NR	1 h in total	NR	None	Yes	CT		NR
Dekalo S, 2019	^68^Ga-PSMA-11	GE/Discovery 690	NR (148-166.5)	45-60	4	NR	None	Yes	CT		No
Fendler WP, 2016	^68^Ga-PSMA-11	Siemens/Biograph 64 TruePoint and GE/Discovery 690	*192 ± 48 (104-276)	*58 ± 12 (45-80)	NR	Yes	None	Yes	CT		Yes
Gao J, 2019	^68^Ga-PSMA-11	United Imaging/uMI 780	131.72 (130.6-177.6)	45	3	NR	None	No	CT		No
Grubmuller B, 2018	^68^Ga-PSMA-11	Siemens/Biograph mMR	2/kg body weight	45	4	Yes	EPE: T2WI assessment in PSMA-positive lesion SVI: PSMA focus present in parts of seminal vesicles	Yes	MRI	3 T, mp	
Gupta M, 2018	^68^Ga-PSMA-11	Siemens/Biograph TruePoint40	2/kg body weight	NR	4	NR	EPE: irregular prostate outline or extraprostatic structure involvement SVI: increased uptake in seminal vesicles.	Yes	CT		NR
Muehlematter UJ, 2019	^68^Ga-PSMA-11	GE/SIGNA	*131 ± 18.8 (98-158)	60	15, prostate 2-3, rest of body	Yes	MRI findings + abnormal uptake outside prostate or in seminal vesicle	Yes	MRI	^c^Mostly 3 T, bp	
Thalgot M, 2018	^68^Ga-PSMA-11	Siemens/Biograph mMR	138 (IQR, 114-156)	55 (IQR, 50-67)	15, prostate 5, rest of body	Yes	EPE = MRI findings SVI = MRI findings + uptake in seminal vesicle	Yes	MRI	3 T, mp	
van Leeuwen PJ, 2019	^68^Ga-PSMA-11	Phillips/Ingenuity and Gemini	^a^2.0/kg ^b^100	^a^60 ^b^45	^a^3 ^b^2 except for 3 in abdomen/pelvis	NR	None	Yes	CT		Yes
von Klot CAJ, 2017	^68^Ga-PSMA-I&T	Siemens/Biograph mCT	*98 ± 25 (60-130)	60 min	Continuous motion at 0.9 mm/s (chest-abdomen), 2.1 mm/s (leg)	NR	EPE: angulated contour of the prostate gland SVI: none	Yes	CT		No
Yilmaz B, 2019	^68^Ga-PSMA-11	Siemens/NR	175 (77-350)	60 min	3	NR	EPE: none SVI: visually as positive or negative	NR	CT		

*BP* Biparametric, *CT* Computed tomography, *Ga* Gallium, *EPE* Extraprostatic extension, *MP* Multi-parametric, *MRI* Magnetic resonance imaging, *NR* Not reported; *PET* Positron emission tomography; *PSA* Prostate-specific antigen, *PSMA* Prostate-specific membrane antigen, *SVI* Seminal vesical invasion

*Data presented in mean ± standard deviation; others are presented in median and ranges

^a^St Vincent’s Hospital

^b^Netherlands Cancer Institute

^c^3 T performed in 36/40 patients and 1.5 T in 4/40

### Quality assessment

**Fig. 2:**
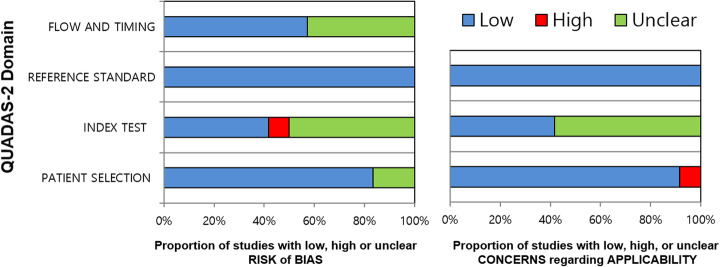
Grouped bar charts for QUADAS-2 tool summarizing risk of bias and concern for applicability of the 12 included studies

All studies were of moderate to good quality, satisfying at 4 or more of the 7 domains in the QUADAS-2 tool except for one which only met 3 domains (Fig. 2). In the patient selection domain, 2 studies had unknown risk of bias as it was not clear whether the patient enrollment was consecutive or not (Agrawal et al. 2017; von Klot et al. 2017). One study had high concern for applicability as minority of the patients (2/50) had rising PSA after radiation treatment and these patients could not be separately analyzed from the other 48 with newly diagnosed prostate cancer (Berger et al. 2018). Regarding the index test domain, six studies had unknown risk of bias and concern for applicability as there were no clear criteria for interpreting SVI and EPE (Agrawal et al. 2017; Berger et al. 2018; Dekalo et al. 2019; Fendler et al. 2016; Gao et al. 2019; van Leeuwen et al. 2019; Yilmaz et al. 2019). One additional study which did not have clear criteria and therefore unclear concern for applicability, had high risk of bias as the interpretation of PSMA-PET was performed without blinding to the surgico-pathological reference standard [30]. In the reference standard domain, all studies were at low risk of bias and concern for applicability. In the flow and timing domain, 6 studies had unknown risk of bias as the interval between PSMA-PET and prostatectomy was not provided (Agrawal et al. 2017; Dekalo et al. 2019; Grubmuller et al. 2018; Gupta et al. 2018; van Leeuwen et al. 2019; von Klot et al. 2017).

### Diagnostic performance of PSMA PET for SVI

**Fig. 3:**
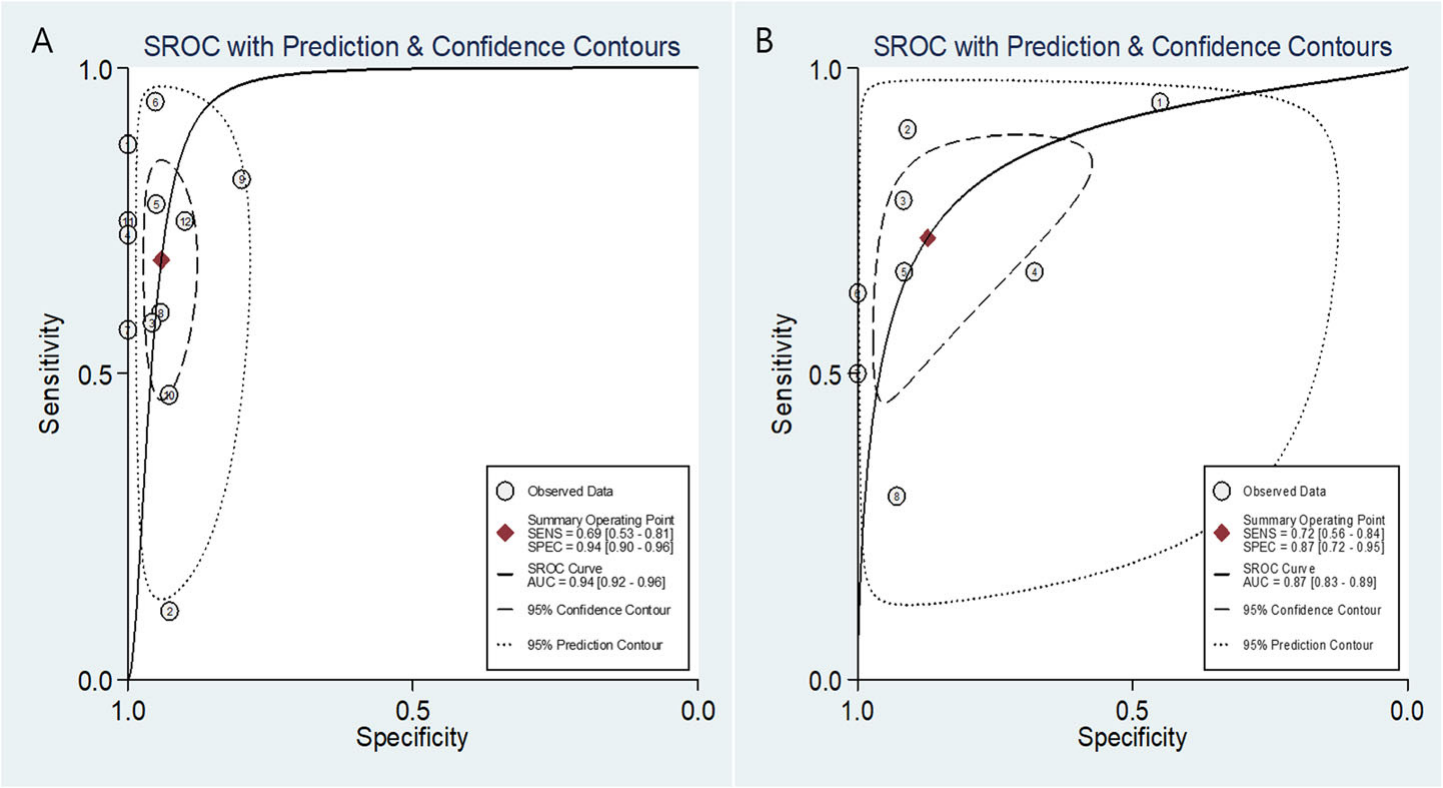
Hierarchical summary receiver operating characteristic curves for PSMA-PET detecting (**a**) seminal vesical invasion and (**b**) extraprostatic extension

**Fig. 4:**
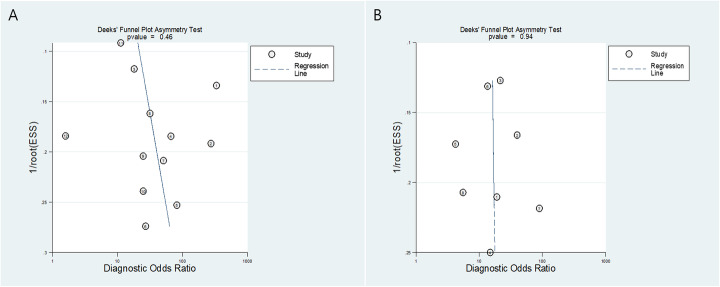
Deeks’ funnel plot. *P* values of 0.46 and 0.94 for studies assessing (**a**) seminal vesical invasion and (**b**) extraprostatic extension indicate absence of publication bias

**Fig. 5:**
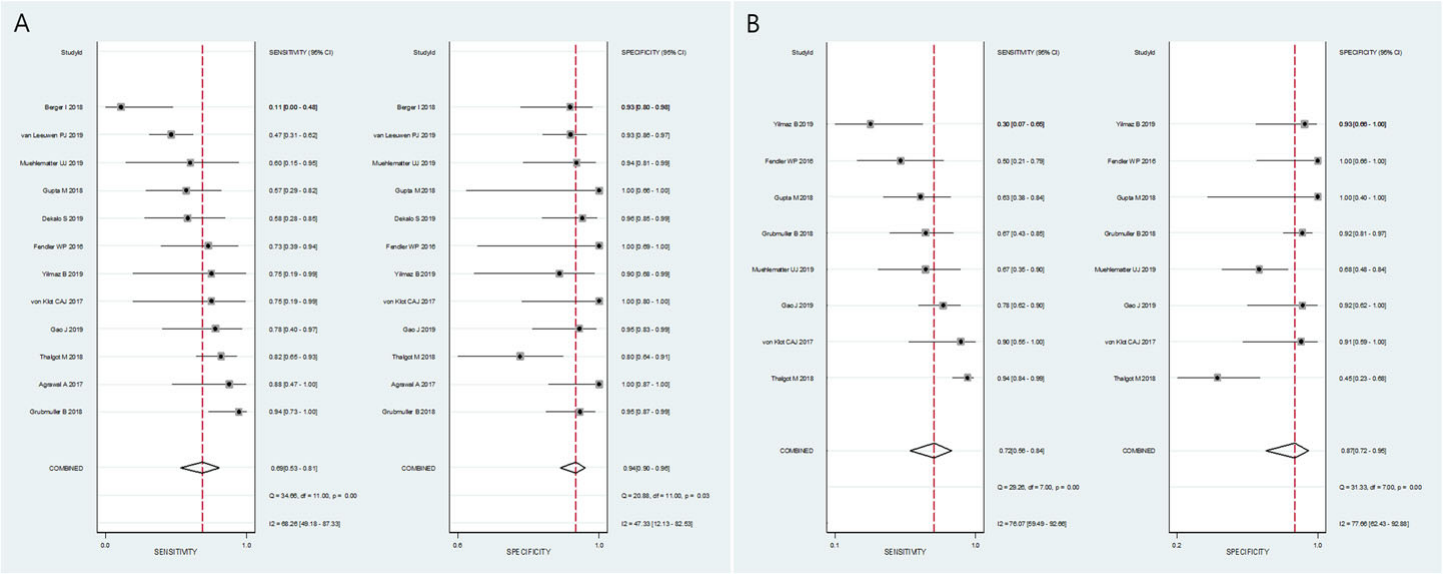
Coupled forest plots of sensitivity and specificity for (**a**) seminal vesical invasion and (**b**) extraprostatic extension. Numbers are pooled estimates with 95% confidence intervals (CI) in parentheses and heterogeneity statistics are shown at the bottom right. Horizontal lines indicate 95% CIs

The summary sensitivity and specificity were 0.69 (95% CI 0.53-0.81) and 0.94 (95% CI 0.90-0.96), respectively (Fig. 3). The area under the HSROC curve was 0.94 (95 % CI 0.92–0.96). No publication bias was suggested in the Deeks’ funnel plot (*p* = 0.46 for slope coefficient) (Fig. 4). The *Q* test indicated that heterogeneity was present (*p* = 0.007), which was substantial and moderate for sensitivity (*I*^2^ = 68%) and specificity (*I*^2^ = 47%), respectively, based on the Higgin’s *I*^2^ test. The coupled forest plot did not show a threshold effect (Fig. 5) with no demonstrable correlation between sensitivity and false-positive rate (correlation coefficient = 0.014 [95% CI, −0.564-0.583]). At meta-regression analysis, anatomical imaging modality was a source of heterogeneity (*p* = 0.02) with PET/MRI showing significantly greater sensitivity (0.87 [95% CI 0.75-0.98]) for detecting SVI compared with PET/CT (0.60 [95% CI 0.47-0.74]) while the specificity was comparable (0.91 [95% CI 0.84-0.97] vs. 0.96 [95% CI 0.93-0.99], respectively).

### Diagnostic performance of PSMA PET for EPE

The summary sensitivity and specificity were 0.72 (95% CI 0.56-0.84) and 0.87 (95% CI 0.72-0.94), respectively (Fig. 3). The area under the HSROC curve was 0.87 (95% CI 0.83–0.89). No publication bias was suggested in the Deeks’ funnel plot (*p* = 0.94 for slope coefficient) (Fig. 4). The *Q* test indicated that heterogeneity was present (*p* < 0.001), which was substantial for both sensitivity (*I*^2^ = 76%) and specificity (*I*^2^ = 78%), respectively. A threshold effect was suggested based on the coupled forest plots (Fig. 5) with a positive correlation between sensitivity and false-positive rate (correlation coefficient = 0.563 [95% CI, −0.234-0.908]). At meta-regression analysis, anatomical imaging modality was not a significant factor of heterogeneity *(*p = 0.08). Studies using PET/MRI demonstrated summary sensitivity and specificity of 0.82 [95% CI 0.67-0.97] and 0.73 [95% CI 0.52-0.94], respectively; whereas for studies using PET/CT, they were 0.65 [95% CI 0.47-0.83] and 0.95 [95% CI 0.89-1.00], respectively.

## Discussion

In the current meta-analysis, we evaluated the diagnostic performance of PSMA-PET in detecting SVI and EPE in patients with newly diagnosed prostate cancer treated with radical prostatectomy. We found that PSMA-PET had moderate sensitivity and excellent specificity for both SVI and EPE. It has already been well recognized in the literature that PSMA-PET shows good performance in detecting and localizing the primary tumor along with its excellent ability to detect metastases in the regional nodes, bones, and soft tissues. The addition of accurate assessment of the local extent of prostate cancer reported herein provides additional rationale for PSMA-PET to be used as a “one-stop-shop” imaging modality in the primary staging of prostate cancer.

There was moderate to substantial heterogeneity among the studies using PSMA-PET to assess SVI. We were able to ascertain that one major source of heterogeneity was whether MRI or CT was used as the anatomical imaging component of PSMA-PET. The three studies (Grubmuller et al. 2018; Muehlematter et al. 2019; Thalgott et al. 2018) using PET/MRI showed significantly greater sensitivity (0.87 vs. 0.60) with similar specificity (0.91 vs. 0.96) compared with the other nine studies (Agrawal et al. 2017; Berger et al. 2018; Dekalo et al. 2019; Fendler et al. 2016; Gao et al. 2019; Gupta et al. 2018; van Leeuwen et al. 2019; Yilmaz et al. 2019) using PET/CT. The superior sensitivity of PSMA-PET/MRI compared with PSMA-PET/CT can be attributed to the synergistic effect of combining the MRI findings with the functional information from PSMA-PET. All three studies using PET/MRI were performed at 3-Tesla scanners with biparametric protocol in one (Muehlematter et al. 2019) and multiparametric protocol in the remaining two studies, which was shown to be helpful for increasing the sensitivity of detecting SVI in a prior meta-analysis by de Rooij et al. (de Rooij et al. 2016). In addition, PET/MRI potentially enhances the detection of the primary tumor itself compared with multiparametric MRI (Eiber et al. 2016; Hicks et al. 2018; Park et al. 2018). On the contrary, CT on its own has a limited role in detecting the tumor and assessing the local extent, rather simply provides an anatomical correlate for assessing the areas of tracer uptake on the PSMA-PET. Nevertheless, it should be noted that there were only three studies using PET/MRI and they were indirectly compared with other studies using PET/CT. Further larger studies performing a head-to-head comparison between PET/MRI and PET/CT are needed to validate the potential superiority of using MRI over CT in determining SVI.

Substantial heterogeneity was also noted among studies assessing EPE, and at least part of this was attributed to a threshold effect. This is an expected finding in diagnostic test accuracy meta-analyses as the sensitivity and specificity both depend on the “threshold” or “cut-off” of determining the positivity of a test. Lowering the threshold on PSMA-PET for determining EPE would theoretically increase sensitivity at the cost of decreased specificity (or increased false-positive rate). Unlike in studies evaluating SVI, the anatomical imaging modality was not a factor of heterogeneity. However, when looking in detail at the subgroup of studies assessing EPE using PET/MRI and PET/CT, the summary sensitivity and specificity estimates were substantially different (0.82 vs. 0.65 and 0.73 vs. 0.95 for PET/MRI vs PET/CT, respectively) with wide and overlapping confidence intervals. These findings, along with the fact that there were only a small number of studies (*n* = 8) evaluating the performance of PSMA-PET for EPE, implicate that strong conclusions cannot be drawn.

The technical details for acquisition of PSMA-PET and its interpretation varied widely among the included studies. First, regarding the PSMA-targeting ligand, most studies used ^68^Ga-PSMA-11, one study used ^68^Ga-PSMA-I&T. Furthermore, although we only assessed ^68^Ga-based radioligands, there are other newer non-^68^Ga-based radioligands, which show promising results. For example, one comparative study for ^18^F–PSMA-1007 and ^68^Ga-PSMA-11 in 16 patients showed that ^18^F–PSMA-1007 may potentially have higher detectability for low-grade cancer (Gleason grade 3) than ^68^Ga-PSMA-11 (Kuten et al. 2020). In another study that included 7 patients, ^18^F–PSMA-1007 PET/MRI yielded a sensitivity and specificity of 100% each for determining SVI. The main difference between ^18^F–PSMA-1007 and ^68^Ga-PSMA-11 is the reduced renal excretion of the ^18^F-labeled compound, a potential benefit for local staging in close proximity to the bladder. Additional PSMA tracers (e.g., ^18^F-DCFBC, ^18^F-DCFPyL) are already currently being used, and as newer tracers are being developed, future studies are needed to investigate whether differences in PSMA tracers will result in different diagnostic capability (Walker et al. 2020). There was also wide variability in the injected radiopharmaceutical dose, uptake, and image acquisition time and usage of diuretics, which can also potentially affect the performance of PSMA-PET (Derlin et al. 2016). Even more importantly, most of the studies did not define set criteria for assessing SVI and EPE regarding each of the components of PSMA-PET and MRI/CT, along with how to perform an integrated interpretation of them. For MRI, several criteria for assessment of EPE (e.g., ESUR criteria, Mehralivand grading system, and length of tumor capsular contact) have been tested and validated in some studies (Mehralivand et al. 2019; Barentsz et al. 2012; Kim et al. 2020). Furthermore, recent efforts by multidisciplinary international group of experts, focused on proposing a standardized assessment of PSMA-PET with the molecular imaging TNM system (miTNM, version 1.0) and PSMA reporting and data systems (PSMA-RADS) version 1.0; however, these systems have neither been tested nor validated in the literature (Eiber et al. 2018; Rowe et al. 2018). Nevertheless, the promising results in the current meta-analysis despite the lack of standardization of image acquisition and interpretation show not only the high potential of PSMA-PET for local staging but also the need for clear and validated criteria for the performance and interpretation of PSMA-PET for local staging. These criteria will need to (1) define the roles of PET and CT/MRI for determining disease extent and to (2) address how these functional and anatomical imaging components can be interpreted together. This could also help improve the inter-reader agreement which was only fair in one of the included studies by Muehleamatter et al. (Muehlematter et al. 2019) that assessed it (kappa of 0.33 for SVI and 0.40 for EPE), and accelerate its widespread adoption.

There are some limitations in this meta-analysis. The number of included studies was small (*n* = 12 for SVI and 8 for EPE, respectively). Notwithstanding, this is currently the largest collective data providing a summary estimate of the performance of PSMA-PET in determining the local extent of disease in prostate cancer in the setting of initial staging. In addition, even with the small number of studies, we were able to identify meaningful sources of heterogeneity that may have clinical implications. Second, all but two were retrospective single-center studies and therefore have the potential of inherent bias. Prospective multicenter studies may be needed to validate the reported diagnostic performance. Third, nearly all patients included had clinically intermediate-to-high risk prostate cancer, and our results may not translate to those with low-risk disease. Fourth, as we used radical prostatectomy as the reference standard, the results are not directly applicable to patients receiving other treatments (e.g., active surveillance, focal treatment, radiation treatment, or systemic treatment). Fifth, EPE was not stratified by extent in all of the included studies. Investigators have observed trends of increasing sensitivity of MRI in detecting EPE with more extensive degrees of EPE. There may be value in designing future studies on PSMA-PET with assessment of EPE and SVI stratified to extent (e.g., < 1 mm, 1–2 mm, and > 2 mm) (Jager et al. 1996; Rosenkrantz et al. 2013).

## Conclusion

PSMA-PET has moderate sensitivity and excellent specificity for assessing the local extent of the tumor in patients with intermediate to high-risk prostate cancer. PET/MRI showed potential for greater sensitivity than PET/CT in assessing SVI. Standardization of image acquisition and interpretation is needed to increase applicability and implementation of our results.

## Data Availability

All data was based on the 12 included papers that are accessible from either the journal webpages or by using Pubmed and EMBASE databases, some of which require journal subscription fees.
